# Dual Use of the METSSS Model Predicting Survival After Palliative Radiotherapy: An Exploratory Analysis

**DOI:** 10.7759/cureus.21223

**Published:** 2022-01-13

**Authors:** Carsten Nieder, Bård Mannsåker, Rosalba Yobuta

**Affiliations:** 1 Oncology, Nordland Hospital Trust, Bodø, NOR

**Keywords:** treatment discontinuation, comorbidity, prognostic model, brain metastases, bone metastases, radiotherapy, palliative radiation therapy

## Abstract

Introduction: The recently published METSSS model, which was developed for prediction of survival after palliative radiotherapy, includes age, sex, cancer type, localization of distant metastases, Charlson-Deyo comorbidity score and radiotherapy site. Its ability to predict other relevant endpoints has not been studied yet. Therefore, this exploratory study analyzed the endpoints “unplanned termination of radiotherapy” and “treatment in the last 30 days of life” in the METSSS-defined risk groups (low/medium/high).

Methods: The risk group was assigned in the METSSS online calculator for our patient cohort with non-hematological malignancies treated between 2009 and 2014 during the first course of treatment (resembling details of the original METSSS study). All patients were treated with classical palliative dose/fractionation regimes such as five fractions of 4 Gy, 10 fractions of 3 Gy or 13 fractions of 3 Gy. No stereotactic high-dose radiation was utilized. Given that single-fraction radiotherapy cannot be discontinued, patients treated with 8 Gy x1 for uncomplicated painful bone metastases were excluded. Both completed and discontinued multi-fraction radiotherapy courses (at least two fractions intended) were included.

Results: The study included 290 patients, 19 of whom failed to complete their prescribed course of palliative radiotherapy (7%). Thirty-nine (13%) were irradiated in the last 30 days of life. Only one patient was classified as low-risk according to the METSSS model (medium: 15, high: 274). Only Eastern Cooperative Oncology Group (ECOG) performance status (PS) was significantly associated with incomplete treatment. All 16 patients with low/medium METSSS risk scores completed their prescribed course of radiotherapy, compared to the 93% completion rate in the high-risk group, p=0.41. With regard to treatment in the last 30 days of life, ECOG PS, metastases to brain, liver and lung, and the number of prescribed fractions were statistically significant. One patient with a low/medium METSSS risk score was treated in the last 30 days of life (6%), compared to 14% in the high-risk group, p=0.49.

Conclusion: Unexpected imbalances in the METSSS risk group size resulted in lower statistical power than anticipated. Patients with low/medium METSSS risk scores performed numerically better. However, other predictive factors, especially ECOG PS, which is not part of the METSSS model, maybe more relevant. Further efforts towards the application of the model beyond its original objective cannot be recommended.

## Introduction

In the context of palliative radiotherapy prescribed to improve cancer-related symptoms, short-course treatment offers a convenient yet efficacious approach [1]. Nevertheless, unplanned termination of short multi-fraction regimens might still occur, mainly as a consequence of the rapid deterioration of patient's performance status (PS) [2,3]. Lack of support and/or financial resources is a less common cause of incomplete radiotherapy and is virtually absent in Norway where the publicly funded healthcare system provides transportation, housing, access to community-based oncology nurses, home care and other services [4,5]. Thus, barriers that were identified in other healthcare regions, e.g. lack of health insurance or high out-of-pocket costs, do not result in socioeconomic disparities. Analyses of predictors of incomplete palliative radiotherapy performed by a Norwegian stakeholder provide useful insights, due to lack of confounding by socioeconomic factors. Under such unique preconditions, factors such as age and disease extent are expected to correlate with the endpoint of incomplete radiotherapy, i.e. factors that also influence overall survival (classic prognostic factors). Prognostic models which include several of these factors, especially models that predict very short survival (treatment in the last 30 days of life) may also provide information about the likelihood of incomplete radiotherapy, and dual-use of such models would be time-saving for busy clinicians who attempt to select the best management approach.

Recently, Zaorsky et al. reported a National Cancer Database analysis and introduced the METSSS model, which identifies patients at greatest risk of death after palliative radiotherapy delivered during the first course of treatment [6]. A nomogram was created and validated to predict survival, available online, https://tinyurl.com/METSSSmodel. The median survival times were 12 months, five months and three months in the low, medium and high-risk groups, respectively. We hypothesized that this model may be useful to predict two other endpoints, i.e. incomplete palliative radiotherapy and treatment in the last 30 days of life. These endpoints were evaluated in a database recently employed to validate the primary outcome of the METSSS model [7].

## Materials and methods

The METSSS model includes age, sex, cancer type (breast, prostate, lung, others), localization of distant metastases (brain, bone, liver, lung), Charlson-Deyo comorbidity score, and radiotherapy site and was calculated online for our patient cohort with non-hematological malignancies treated between 2009 and 2014 during the first course of treatment, i.e. no re-irradiation or repeat irradiation. These patients were treated with classical palliative dose/fractionation regimes such as five fractions of 4 Gy, 10 fractions of 3 Gy or 13 fractions of 3 Gy. No stereotactic high-dose radiation was utilized. Given that single-fraction radiotherapy cannot be discontinued, patients treated with 8 Gy x1 for uncomplicated painful bone metastases were excluded. Both completed and discontinued multi-fraction radiotherapy courses (at least two fractions intended) were included without restricting indication, treated body region and a number of treated target volumes. Radiation was either administered to the primary tumor or metastatic sites. All patients were managed with standard-of-care systemic therapy if indicated.

All parameters required to employ the METSSS calculator were available for all patients (no missing data). According to their predicted survival, patients were classified as low, medium or high risk. Due to group sizes, low and medium were combined. In the two resulting groups, the rate of permanent discontinuation and administration of radiotherapy in the last 30 days of life was calculated. Two-tailed Fisher exact probability tests were employed to compare the rates (SPSS 27; IBM Corp., Armonk, NY, USA). Statistical significance was defined as p<0.05. Our database, which was created for the purpose of quality-of-care analyses, does not require additional approval by the local Ethics Committee (REK Nord) for secondary evaluations like the present one.

## Results

This study included 290 patients, 19 of whom failed to complete their prescribed course of palliative radiotherapy (7%). Thirty-nine (13%) were irradiated in the last 30 days of life. The baseline characteristics are shown in Table 1.

**Table 1:** Baseline data (n=290). *Eastern Cooperative Oncology Group PS - performance status

Baseline parameter	Number	Percent
Gender		
Female gender	109	38
Male gender	181	62
Cancer type		
Prostate cancer	56	19
Breast cancer	41	14
Lung cancer	93	32
Colorectal cancer	23	8
Kidney cancer	26	9
Other solid cancer	51	18
Site of metastases		
Brain metastases	63	22
Liver metastases	48	17
Lung metastases	80	28
Bone metastases	163	56
Radiation to metastatic sites		
Irradiated for bone metastases	162	56
Irradiated for brain metastases	52	18
Irradiated for lymph node metastases	35	12
Irradiated to lung/mediastinum	49	17
Irradiated to bladder/prostate	9	3
Irradiated to other targets	9	3
ECOG performance status*		
PS 0-1	147	51
PS 2	86	30
PS 3-4	57	20
Comorbidity		
Charlson-Deyo score 0	114	39
Charlson-Deyo score 1	66	23
Charlson-Deyo score 2	65	22
Charlson-Deyo score >2	45	16
Radiation regimen		
Max. 5 fractions prescribed	64	22
6-10 fractions prescribed	162	56
More than 10 fractions prescribed	64	22
Age (years)		
Mean (standard deviation), range	68 (11), 23-92	

Regarding cancer type, lung cancer (32%) was more common than prostate (19%) or breast cancer (14%). Fifty-six percent were irradiated for bone metastases. Prescription of 6-10 fractions was common (56%). Only one patient was classified as low-risk according to the METSSS model (medium: 15, high: 274). Median survival was 5.3 (high) and 17.6 months (medium), respectively (p=0.03).

With regard to incomplete radiotherapy, all baseline parameters displayed in Table 1 were analyzed. As indicated in Table 2, only Eastern Cooperative Oncology Group (ECOG) PS was significantly associated with this endpoint.

**Table 2:** Risk factors for incomplete radiotherapy and irradiation in the last 30 days of life. *Eastern Cooperative Oncology Group n.s. not significant, p>0.05

Parameter	Percent incomplete	P-value	Percent last 30 days of life	P-value
Brain metastases			24	
No brain metastases		n.s.	11	0.01
Liver metastases			33	
No liver metastases		n.s.	10	<0.001
Lung metastases			23	
No lung metastases		n.s.	10	0.01
Performance status 0-1*	1		1	
Performance status 2	7		15	
Performance status 3-4	19	<0.001	42	<0.001
Max. 5 fractions prescribed			24	
6-10 fractions prescribed			12	
More than 10 fractions prescribed		n.s.	5	0.005

All 16 patients with low/medium METSSS risk scores completed their prescribed course of radiotherapy, compared to the 93% completion rate in the high-risk group, p=0.41.

With regard to treatment in the last 30 days of life, the same set of baseline parameters was analyzed. Besides ECOG PS, metastases to brain, liver and lung, and the number of prescribed fractions were statistically significant. One patient with a low/medium METSSS risk score was treated in the last 30 days of life (6%), compared to 14% in the high-risk group, p=0.49. The single most noticeable factor was ECOG PS 3-4, where 42% of the patients received radiotherapy in the last 30 days of life.

## Discussion

This study was performed to analyze the ability of the METSSS model to predict two other relevant endpoints, i.e. incomplete palliative radiotherapy and treatment in the last 30 days of life. With 290 eligible patients treated between 2009 and 2014 (identical to the time period in the METSSS study [6]), the study was not particularly small. However, surprisingly many patients (94%) were classified as high-risk and thus, the statistical power of the main comparisons (low/medium versus high-risk) was much lower than expected. The numerical differences (low rates of incomplete treatment and irradiation in the last 30 days of life in the small group with low/medium risk), open for the possibility of statistically significant differences in a study with several thousands of patients, like the original METSSS study. However, efforts to perform large-scale analyses might not be warranted, given that other parameters, in particular ECOG PS, showed a much larger impact than the METSSS model. Of course, the METSSS model was not developed to primarily address incomplete palliative radiotherapy and treatment in the last 30 days of life, but due to its excellent discrimination of survival outcomes and simplicity, we felt that an exploratory analysis of these two clinically meaningful endpoints still could provide informative insights. A time-saving model that could provide multiple outcome measures would probably gain wider clinical acceptance. Interestingly, several components of the METSSS model, e.g. age, sex, cancer type, and comorbidity, were not significantly associated with the two endpoints. This fact again explains why other parameters or models appear more promising than METSSS.

Furthermore, due to the utilization of the National Cancer Database, which captures treatment delivered during the first course only, the METSSS model may have limitations during the terminal stage of the disease. Compared to older models such as TEACHH [8,9], METSSS includes a comorbidity score (a parameter that has been tied to overall survival also previously [10]). On the other hand, PS is not included, despite a large body of evidence, which has demonstrated its tremendous impact on survival [8-10]. In the present study, PS was associated with failure to complete radiotherapy and administration of radiation in the last 30 days of life (Table 2). Interestingly, not all predictors of radiation in the last 30 days of life were significantly associated with failure to complete radiotherapy. Age was not among the relevant parameters, meaning that also geriatric patients should be considered for palliative radiotherapy.

In general, radiotherapy providers should try to achieve the goals of palliative treatment without causing unnecessary burden, both regarding side effects, costs and inconvenience [11-13]. Quality measures such as incomplete radiotherapy and treatment in the last 30 days of life can be monitored, both on longitudinal institutional and larger levels [14,15]. According to a review by Park et al. [15], the overall palliative radiotherapy utilization rates during the last month of life were in the range of 9%-15%. The most commonly used regimen was 30 Gy in 10 fractions (36%-90%). ECOG PS 3-4 was significantly associated with patients receiving radiotherapy in the last 30 days of life and shorter survival. Twenty-six percent of patients who survived less than one month were reported to show symptom palliation following radiotherapy. A more recent study by Vázquez et al. included 708 patients to whom 992 palliative treatment courses were delivered [16]. The most frequent primary tumor site was the lung (31%). Bone was the predominant target volume of the treatment (56%). The 30-day mortality was 17.5%. In the multivariate analysis, male gender, ECOG PS 2-3, gastrointestinal and lung cancer were found to be independent factors related to 30-day mortality.

Kain et al. studied 1,744 patients [17]. Thirty-day mortality was 10% and was higher in patients with lung cancer, patients having less than five fractions, and patients in TEACHH group B/C. Lee et al. developed a predictive score for 30-day mortality after palliative radiotherapy in more than 5000 patients with metastatic cancer receiving first-course palliative radiotherapy [18]. Seventeen percent died within 30 days of radiotherapy commencement. The most important mortality predictors were primary lung cancer and log peripheral blood neutrophil-lymphocyte ratio. The developed predictive scoring system had 10 predictor variables including several blood test results. Inclusion of these is a potential advantage compared to simpler models without assessment of blood tests, as recently also shown in other studies evaluating overall survival [19]. On the other hand, limiting inclusion to patients with stage IV cancer and first-course palliative radiotherapy does not resemble the complete clinical picture.

Despite major advances in our understanding of factors that indicate potentially futile radiotherapy, at least in terms of short survival and a limited number of days with improved symptoms before death, if any, the search for universally agreed models or decision tools continues. In addition to PS, cancer type, disease burden/location of metastases, blood test results, eligibility for systemic therapy and other parameters are components of currently available models. Discussions between patients and care providers, which often involve more than one medical discipline and profession, might be facilitated by the utilization of validated scores or nomograms, which add transparency and reproducibility in the complex scenario of defining treatment goals and priorities near the end of life.

## Conclusions

Unexpected imbalances in the METSSS risk group size resulted in lower statistical power than anticipated. Patients with low/medium METSSS risk scores performed numerically better. However, other predictive factors, especially ECOG PS, which is not part of the METSSS model, maybe more relevant. Further efforts toward the application of the model beyond its original objective cannot be recommended.
